# A Genetic Validation Study Reveals a Role of Vitamin D Metabolism in the Response to Interferon-Alfa-Based Therapy of Chronic Hepatitis C

**DOI:** 10.1371/journal.pone.0040159

**Published:** 2012-07-12

**Authors:** Christian M. Lange, Stephanie Bibert, Zoltan Kutalik, Philippe Burgisser, Andreas Cerny, Jean-Francois Dufour, Andreas Geier, Tilman J. Gerlach, Markus H. Heim, Raffaele Malinverni, Francesco Negro, Stephan Regenass, Klaus Badenhoop, Jörg Bojunga, Christoph Sarrazin, Stefan Zeuzem, Tobias Müller, Thomas Berg, Pierre-Yves Bochud, Darius Moradpour, the Swiss Hepatitis C Cohort Study Group

**Affiliations:** 1 Division of Gastroenterology and Hepatology, Centre Hospitalier Universitaire Vaudois, University of Lausanne, Lausanne, Switzerland; 2 Medizinische Klinik 1, Klinikum der J. W. Goethe-Universität Frankfurt a.M., Frankfurt a.M., Germany; 3 Division of Infectious Diseases, Centre Hospitalier Universitaire Vaudois, University of Lausanne, Lausanne, Switzerland; 4 Division of Medical Genetics, Centre Hospitalier Universitaire Vaudois, University of Lausanne, Lausanne, Switzerland; 5 Liver Unit, Ospedale Moncucco, Lugano, Switzerland; 6 University Clinic of Visceral Surgery and Medicine, Inselspital, Bern, Switzerland; 7 Division of Gastroenterology and Hepatology, University Hospital Zürich, Zürich, Switzerland; 8 Division of Gastroenterology, Kantonsspital St. Gallen, St. Gallen, Switzerland; 9 Division of Gastroenterology and Hepatology, University Hospital Basel, Basel, Switzerland; 10 Hôpital Neuchâtelois, Neuchâtel, Switzerland; 11 Division of Gastroenterology and Hepatology, University Hospital Geneva, Geneva, Switzerland; 12 Medizinische Klinik mit Schwerpunkt Hepatologie und Gastroenterologie, Charité Campus Virchow Klinikum, Berlin, Germany; 13 Klinik für Gastroenterologie und Rheumatologie, Sektion Hepatologie, Universitätsklinikum Leipzig, Leipzig, Germany; University of Washington, United States of America

## Abstract

**Background:**

To perform a comprehensive study on the relationship between vitamin D metabolism and the response to interferon-α-based therapy of chronic hepatitis C.

**Methodology/Principal Findings:**

Associations between a functionally relevant polymorphism in the gene encoding the vitamin D 1α-hydroxylase (*CYP27B1-1260* rs10877012) and the response to treatment with pegylated interferon-α (PEG-IFN-α) and ribavirin were determined in 701 patients with chronic hepatitis C. In addition, associations between serum concentrations of 25-hydroxyvitamin D_3_ (25[OH]D_3_) and treatment outcome were analysed. *CYP27B1-1260* rs10877012 was found to be an independent predictor of sustained virologic response (SVR) in patients with poor-response *IL28B* genotypes (15% difference in SVR for rs10877012 genotype AA *vs*. CC, p = 0.02, OR = 1.52, 95% CI = 1.061–2.188), but not in patients with favourable *IL28B* genotype. Patients with chronic hepatitis C showed a high prevalence of vitamin D insufficiency (25[OH]D_3_<20 ng/mL) during all seasons, but 25(OH)D_3_ serum levels were not associated with treatment outcome.

**Conclusions/Significance:**

Our study suggests a role of bioactive vitamin D (1,25[OH]_2_D_3_, calcitriol) in the response to treatment of chronic hepatitis C. However, serum concentration of the calcitriol precursor 25(OH)D_3_ is not a suitable predictor of treatment outcome.

## Introduction

Chronic hepatitis C is one of the most serious infectious diseases worldwide [1]. Less than 50% of all patients infected with hepatitis C virus (HCV) genotype 1 and 4 as well as ∼80% of those infected with genotype 2 and 3 can be cured with a combination therapy of pegylated interferon-α (PEG-IFN-α) and ribavirin [2]. The adjunction of directly acting antivirals (DAA), namely the NS3-4A protease inhibitors telaprevir and boceprevir, results in substantially increased rates of sustained virologic response (SVR) in both treatment-naïve and treatment-experienced patients infected with HCV genotype 1 [3]–[7]. However, such triple therapy regimens are burdened with additional adverse events and their efficacy in prior null-responders (<2 log_10_ reduction in HCV RNA after 12 weeks of PEG-IFN-α and ribavirin) remains limited [7], [8]. Therefore, despite enormous progress, there is still a need to optimize IFN-α-based (or IFN-α-free) treatment regimens for chronic hepatitis C, and the establishment of algorithms (including, for example, on-treatment viral kinetics and *IL28B* genotype) to select appropriate treatment regimens for individual patients remains highly relevant [9]–[15]. The importance of host genetics in the prediction of treatment outcome has been impressively demonstrated by the discovery of the *IL28B* locus as determinant of spontaneous as well as of treatment-induced clearance from HCV infection [11], [13]. The minor allele of *IL28B* (e. g. rs12979860 T allele) has a an adverse effect on both spontaneous and treatment-induced clearance, and it was shown that for example the adverse *IL28B* rs12979860 CT/TT genotype is one of the strongest baseline predictors of treatment failure [14].

Recently, vitamin D insufficiency (defined by a 25-hydroxyvitamin D [25(OH)D_3_] serum concentration <20 ng/mL) has been proposed as a predictor of failure of treatment of chronic hepatitis C with PEG-IFN-α and ribavirin [16], [17]. Moreover, severe vitamin D deficiency is a common feature of chronic hepatitis C, even in the absence of advanced liver fibrosis [18]. These findings may have important implications for the management of chronic hepatitis C, as vitamin D status is a potentially modifiable determinant of treatment outcome. However, it is currently unknown whether vitamin D itself affects response to IFN-α-based therapy or whether it is only a surrogate marker of treatment outcome.

A number of genetic polymorphisms in the vitamin D pathway have been shown to affect vitamin D signaling, and stratification according to such polymorphisms has already being implemented in randomized controlled clinical intervention studies [19]–[22]. Therefore, we believe that analyzing the impact of functionally relevant genetic polymorphisms in the vitamin D cascade on SVR may provide stronger evidence on an intrinsic role of vitamin D metabolism in the pathogenesis and treatment of chronic hepatitis C than analyzing exclusively vitamin D serum levels, which are affected by various parameters, including season, sunlight exposure, nutrition, and the metabolic syndrome [23], [24]. Recently, some of us have observed an association of the 1α-hydroxylase promoter polymorphism *CYP27B1-1260* rs10877012 with SVR in a relatively small group of patients (n = 110) [18]. In the present study, we aimed to validate this association in 701 patients selected from a well-characterized patient cohort, the Swiss Hepatitis C Cohort Study (SCCS). In addition, we further characterized the relationship between 25(OH)D_3_ serum levels and chronic hepatitis C as well as its treatment.

**Table 1:** Baseline and demographic characteristics.

	Response to therapy		
	SVR (n = 449)	Non-Response (n = 252)	25(OH)D3 serum concentration (n = 496)
***Sex***			
Male, n (%)	276 (61)	170 (67)	314 (63)
Missing, n	0	0	0
***Age, years***			
Mean (range)	44 (23–78)	46 (21–70)	45 (21–76)
Missing, n	79	53	73
***HCV genotype, n (%)***			
1	140 (31)	170 (68)	203 (47)
2	70 (16)	11 (4)	44 (10)
3	206 (46)	43 (17)	148 (34)
4	32 (7)	26 (10)	41 (9)
Missing	1	2	60
***Diabetes, n (%)***			
Yes	26 (6)	26 (10)	32 (6)
Missing	0	0	0
***HCV RNA, log_10_ IU/mL***			
Mean (range)	5.77 (1–8)	5.00 (1–8)	5.66 (1–8)
Missing, n	0	0	3
***Fibrosis stage, n (%)***			
0	27 (9)	12 (7)	31 (10)
1	77 (26)	31 (18)	95 (31)
2	99 (33)	58 (34)	79 (26)
3	36 (12)	33 (19)	44 (15)
4	57 (19)	38 (22)	54 (18)
Missing	153	80	193
***Steatosis, n (%)***			
Yes	221 (72)	122 (76)	208 (73)
Missing, n	144	91	210
***Activity, n (%)***			
None-mild	227 (77)	137 (79)	237 (78)
Moderate-high	67 (23)	36 (21)	66 (22)
Missing, n	155	79	193
***BMI, kg/m^2^***			
Mean (range)	24 (16–42)	25 (18–40)	24 (16–40)
Missing, n	106	70	171
***ALT, U/L***			
Mean (range)	94 (8–693)	107 (18–617)	121 (8–720)
Missing, n	106	70	171

Univariate P values for SVR *vs.* nonresponse are shown in Table 2. ALT, alanine aminotransferase; BMI, body mass index; HCV, hepatitis C virus; SVR, sustained virologic response. Of the 496 patients in whom 25(OH)D3 serum concentrations were measured, 269 were subsequently treated with pegylated interferon-α and ribavirin and assessed for treatment outcome.

## Methods

### Objectives

The primary objective of the present study was to evaluate whether *CYP27B1-1260* rs10877012 genotype, a genetic marker of biologically active vitamin D, is associated with outcome of IFN-α based therapy of chronic hepatitis C. Secondary objectives were to characterize the frequency and determinants of vitamin D deficiency in patients with chronic hepatitis C, and whether serum levels of the calcitriol precursor 25(OH)D_3_ are suitable for the prediction of treatment outcome as well.

**Table 2:** Genotype frequencies of the 1α-hydroxylase promoter polymorphism *CYP27B1-1260* rs10877012 in patients who received treatment with pegylated interferon-α and ribavirin.

	All, n (%)*	HCV gt 1/4, n (%)	HCV gt 2/3, n (%)
AA	63 (9)	34 (9)	29 (9)
CA	293 (42)	153 (42)	139 (42)
CC	345 (49)	181 (49)	162 (49)

The distribution of homozygous and heterozygous carriers corresponded to the expectations from the Hardy-Weinberg equilibrium (P = 0.35).

* This includes 3 patients with unknown HCV genotype. gt, genotype; HCV, hepatitis C virus.

### Participants

Patients were followed within the framework of the SCCS, a multicenter study pursued at 8 major Swiss hospitals and their local affiliated centers, including a total of 3,648 patients with chronic or resolved HCV infection [25]. For the present retrospective analysis, two primary variables were analyzed: outcome of treatment with PEG-IFN-α and ribavirin (SVR *vs*. failure to achieve SVR) and 25(OH)D_3_ serum concentration (as continuous variable). Patients were included in the analysis of treatment response if they had treatment-naïve chronic hepatitis C, had provided written informed consent for genetic testing, had genomic DNA available for testing, were treated under clinical practice conditions with either PEG-IFN-α2a or PEG-IFN-α2b in combination with weight-based ribavirin, with standard treatment durations (48 weeks for HCV genotype 1 and 4, 24 weeks for HCV genotype 2 and 3), and if they had received ≥80% of the recommended dose of both agents during the first 12 weeks of therapy. Patients who received antiviral therapy not according to these pre-defined criteria were excluded from the analyses. Serum concentrations of 25(OH)D_3_ were determined in all patients in whom a plasma sample at baseline of antiviral therapy was available. Moreover, 25(OH)D_3_ serum levels were determined in additional patients from the SCCS in whom a plasma sample at the time of a liver biopsy was available.

**Table 3:** Factors associated with sustained virologic response.

Variable	P value, univariate	P value, multivariate	Odds ratio (95% CI), multivariate
Age (years, continuous)	<0.001	<0.001	0.96 (0.94–0.98)
HCV RNA (log_10_ IU/mL, continuous)	<0.001	<0.001	0.38 (0.29–0.50)
ALT (U/L, continuous)	0.05		
BMI (kg/m^2^, continuous)	0.08		
Male sex	0.10	1.0	0.85 (0.57–1.28)
HCV genotype 2/3 *vs.* 1/4	<0.001	<0.001	8.99 (4.21–19.19)
Diabetes	0.03		
Fibrosis, F0–F1 *vs*. F2–F4	0.03		
Steatosis	0.47		
Necroinflammatory activity	0.71		
Alcohol (40 g/d ≥5 years)	0.28		
*IL28B* rs12979860	<0.001	<0.001	0.27 (0.17–0.43)
*CYP27B1* rs10877012	0.11	0.06	1.33 (0.99–1.80)

ALT, alanine aminotransferase; BMI, body mass index; CI, confidence interval; HCV, hepatitis C virus.

**Figure 1:**
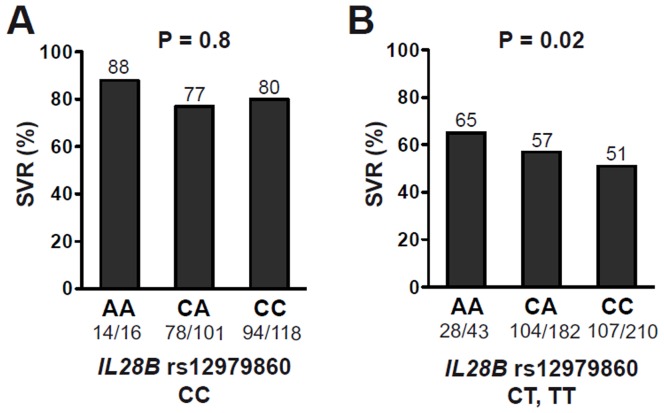
Treatment response according to *CYP27B1-1260* rs10877012 genotype. Overall sustained virologic response (SVR) rates across all HCV genotypes are shown for patients (**A**) with *IL28B* rs12979860 genotype CC and for (**B**) patients with *IL28B* rs12979860 genotype CT or TT. Numbers above bars indicate SVR rates in percent. Numbers below bars indicate absolute numbers of patients with SVR/all patients.

**Table 4:** Factors associated with sustained virologic response in patients with unfavorable *IL28B* rs12979860 genotype CT or TT.

Variable	P value, univariate	P value, multivariate	Odds ratio (95% CI), multivariate
Age (years, continuous)	<0.001	0.001	0.96 (0.94–0.98)
HCV RNA (log_10_ IU/mL, continuous)	<0.001	<0.001	0.35 (0.25–0.50)
ALT (U/L, continuous)	0.10		
BMI (kg/m^2^, continuous)	0.16		
Male sex	0.11	0.11	0.97 (0.59–1.60)
HCV genotype 2/3 *vs.*1/4	<0.001	<0.001	12.4 (5.01–30.9)
Diabetes	0.05		
Fibrosis, F0–F1 *vs*. F2–F4	0.001		
Steatosis	0.82		
Necroinflammatory activity	0.86		
Alcohol (>40 g/d ≥5 years)	0.30		
*CYP27B1* rs10877012	0.06	0.02	1.52 (1.06–2.19)

ALT, alanine aminotransferase; BMI, body mass index; CI, confidence interval; HCV, hepatitis C virus.

Demographic and clinical characteristics including age, sex, HCV genotype, HCV viral load, histological grade and stage, alanine aminotransferase (ALT) serum levels, chronic hepatitis C treatment, alcohol consumption, body mass index (BMI), and presence or absence of diabetes were extracted from the SCCS database. SVR was defined as HCV RNA below the limit of detection in a sensitive assay ≥24 weeks after treatment completion, and all patients who failed to achieve SVR were classified as nonresponders. Data on rapid, early, and end-of-treatment virologic response were not available. High alcohol intake was defined as consumption >40 g per day over a period of ≥5 years. Liver biopsies were evaluated by experienced local pathologists. Liver fibrosis was classified according to the METAVIR score. Necroinflammatory activity was stratified into two groups, absent to mild activity *vs*. moderate to high activity. Steatosis was classified as absent or present.

**Figure 2:**
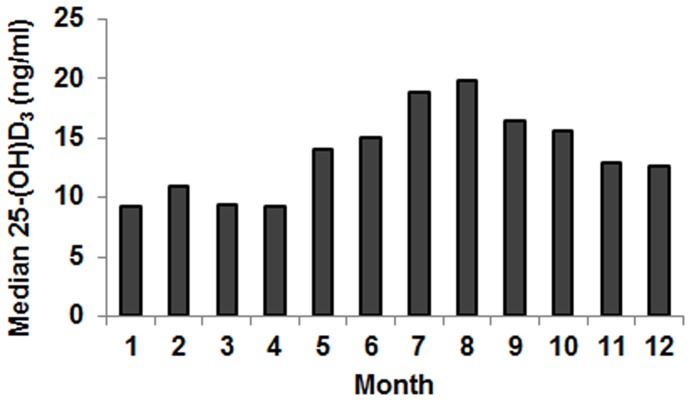
Seasonal variation of 25(OH)D_3_ serum levels in patients with chronic hepatitis C. Median 25(OH)D_3_ serum levels are shown for each month.

### Description of Investigations Undertaken

Serum levels of 25(OH)D_3_ were measured as described previously [18]. Concentrations <20 ng/mL and <10 ng/mL were defined as vitamin D insufficiency and deficiency, respectively, whereas concentrations ≥20 ng/mL were considered as normal [24].

Genotyping for the *CYP27B1-1260* rs10877012 single nucleotide polymorphism (SNP) was performed using a TaqMan SNP Genotyping Assay (Applied Biosystems, Foster City, CA) and the ABI 7500 Fast Real-Time thermocycler, according to manufacturer’s recommendations. TaqMan probes and primers were designed and synthesized by Applied Biosystems: rs10877012 forward 5′-AACAGAGAGAGGGCCTGTCT-3′, reverse 5′-GGGAGTAAGGAGCAGAGAGGTAAA-3′, Vic probe 5′-CTGTGGGAGATTCTTTTAT-3′, Fam probe 5′-TGTGGGAGATTATTTTAT-3′. Automated allele calling was performed using SDS Software from Applied Biosystems. Positive and negative controls were used in each genotyping assay. *IL28B* rs12979860 genotyping was performed as described [26].

**Table 5:** Factors associated with 25-hydroxyvitamin D serum levels (≥ median) in patients with chronic hepatitis C.

Variable	P value, univariate	P value, multivariate	Odds ratio (95% CI), multivariate
Age (years, continuous)	0.2	0.013	1.06 (1.01–1.12)
HCV RNA (log_10_ IU/mL, continuous)	0.3		
ALT (U/L, continuous)	0.6		
BMI (kg/m^2^, continuous)	0.4		
*IL28B* rs12979860	0.3		
Male sex	0.003	0.5	0.77 (0.33–1.80)
HCV genotype 1,2,4 *vs*. 3^1^	0.034		
HIV coinfection	0.6		
Diabetes	0.049	0.01	0.17 (0.04–0.66)
Alcohol (>40 g/d ≥5 years)	0.010		
Fibrosis, F0–F1 *vs*. F2–F4	0.009	0.018	0.31 (0.12–0.82)
Necroinflammatory activity	0.9		
Steatosis	0.07		
Season of blood sampling	<0.0001	<0.0001	6.49 (2.71–15.6)

1 Genotype 1, 2 and 4 were grouped *vs*. genotype 3 in this analysis, as the latter has been associated with metabolic disturbances (insulin resistance, steatosis) and more rapid fibrosis progression [37], [38]. ALT, alanine aminotransferase; BMI, body mass index; CI, confidence interval; HCV, hepatitis C virus.

### Ethics

The study was approved by local ethical committees of each hospital (Universitätsspital Basel, Basel; Inselspital, Bern; University Hospital Geneva, Geneva; CHUV, Lausanne; Ospedale Moncucco, Lugano; Hôpital Neuchâtelois, Neuchatel; Kantonsspital St. Gallen, St. Gallen; Universitätsspital Zürich, Zürich), and written informed consent was received from all participants.

### Statistical Analyses

Testing for Hardy-Weinberg equilibrium was performed with the genhw package in Stata (version 9.1, StataCorp, College Station, TX). Associations of *CYP27B1-1260* rs10877012 SNP with dichotomic variables (SVR *vs.* nonresponse) were assessed in logistic regression models. After univariate analyses, multivariate analyses were performed for significant associations. Multivariate models were obtained by backward selection, using a *P* value >0.15 for removal from the model. Sex, age, and *CYP27B1-1260* rs10877012 genotype were forced into the model. Only patients with complete data for the remaining covariates were included in multivariate analyses. SNPs were analyzed using an additive model (none, one or two copies of the minor allele were coded 0, 1 and 2, respectively, assuming greater effect with increased copy number of the minor allele), unless otherwise specified. Group differences (e.g. *CYP27B1-1260* rs10877012 AA *vs*. AC *vs*. CC) were assessed by means of χ^2^ contingency tables or Wilcoxon-Mann-Whitney-U-tests, as appropriate.

## Results

### Patient Characteristics

Out of a total of 3,648 patients enrolled in the SCCS, 701 patients were included in the analysis of treatment response based on the selection criteria defined above. Of these, 269 patients had a plasma sample at baseline of antiviral therapy available for measurement of 25(OH)D_3_ level. Moreover, 25(OH)D_3_ serum concentrations were determined in an additional 227 patients from the SCCS in whom a plasma sample at the time of a liver biopsy was available. Thus, 25(OH)D_3_ serum levels were determined in a total of 496 patients. Epidemiological and clinical characteristics of patients are summarized in Table 1.

### *CYP27B1-1260* rs10877012 and Response to Treatment of Chronic Hepatitis C

The *CYP27B1-1260* rs10877012 genotype was chosen as the primary variable to be assessed for associations with treatment outcome because this functional polymorphism in the promoter of the 1α-hydroxylase affects tissue concentrations of calcitriol (the C allele results in reduced calcitriol synthesis), thereby being a well-characterized determinant of biologically active vitamin D [20], [27]. rs10877012 genotype frequencies of the present cohort are shown in Table 2. They were largely comparable to those previously observed in the general population [18].

In a pooled analysis across all HCV genotypes, there was a progressive decrease in SVR rates according to *CYP27B1-1260* rs10877012 genotype (73% SVR for genotype AA, 65% for AC and 62% for CC), suggesting an additive effect of the SNP. However, the association was not significant (P = 0.11, OR = 1.22, 95% CI = 0.954–1.552). Significance level increased in a multivariate model, after adjustment for HCV genotype, age, sex, baseline viral load, BMI, ALT, diabetes, and *IL28B* rs12979860 genotype (P = 0.06, OR = 1.33, CI = 0.988–1.796, Table 3) or when comparing rs10877012 CC carriers to AA carriers (P = 0.04). When the association of *CYP27B1-1260* rs10877012 with SVR was examined in patients stratified according to *IL28B* rs12979860 genotype CT and TT *vs.* CC, as shown in Figure 1, *CYP27B1-1260* rs10877012 was an independent predictor of SVR in patients with the poor-response genotype CT or TT (65% *vs*. 57% *vs*. 51% SVR in *CYP27B1-1260* rs10877012 AA *vs*. AC *vs*. CC, respectively; univariate P = 0.06; multivariate P = 0.02, OR = 1.52, 95% CI = 1.061–2.188, Table 4) but not in those with the good-response genotype CC (P = 0.8).

A similar impact of *CYP27B1-1260* rs10877012 on SVR was observed when patients infected with genotype 1 and 4 were analysed exclusively. Again, *CYP27B1-1260* rs10877012 genotype had no significant influence on SVR in patients with good-response *IL28B* rs12979860 genotype (SVR rates 88% *vs.* 65% *vs*. 73% for AA *vs*. CA *vs.* CC, respectively; P = 0.62). However, SVR rates in HCV genotype 1 and 4 patients with poor-response *IL28B* rs12979860 genotype were 42% *vs*. 39% *vs.* 30% in *CYP27B1-1260* rs10877012 AA *vs*. AC *vs*. CC, respectively (univariate P = 0.11; multivariate P = 0.09, OR = 1.46, 95% CI = 0.940–2.288), but the association formally lost statistical significance, most likely due to the low number (n = 24) of HCV genotype 1 and 4 patients with *CYP27B1-1260* rs10877012 AA and poor-response *IL28B* rs12979860 genotype. With respect to the low frequency of the beneficial minor A allele, *CYP27B1-1260* rs10877012 does not appear to be a suitable parameter for clinical decision making. Nevertheless, our genetic analyses point to a relevant role of vitamin D metabolism in the response to treatment with PEG-IFN-α and ribavirin of chronic hepatitis C.

### Serum Concentrations of 25-hydroxyvitamin D in Patients with Chronic Hepatitis C

Serum concentrations of 25(OH)D_3_ were determined in 496 patients at baseline of treatment with PEG-IFN-α and ribavirin (n = 269) or at the time of liver biopsy in patients who were not treated (n = 227). Mean and median 25(OH)D_3_ serum concentrations were 15.6 and 13.4 ng/mL (SD = 9.07; range 3.8–76.9 ng/mL), respectively, which are significantly lower than those determined in the general population (e. g. 22.2 and 20 ng/mL in Reusch et al., n>6.000) [24], [28]. Among patients with chronic hepatitis C, 32%, 42%, and 26% had 25(OH)D_3_ serum levels <10, ≥10 to <20, and ≥20 ng/mL, respectively. Because of the seasonal fluctuation of 25(OH)D_3_ serum levels, we calculated median 25(OH)D_3_ serum levels separately for each month. Although season had a substantial impact on median 25(OH)D_3_ serum levels, at least 50% of HCV-infected individuals suffered from vitamin D insufficiency (<20 ng/mL) even during summer, while approximately 50% suffered from vitamin D deficiency (<10 ng/mL) between November and April (Figure 2).

In a univariate analysis, age, male sex, infection with HCV genotype 3, excessive alcohol consumption, presence of diabetes, advanced liver fibrosis, and season of blood sampling were significantly associated with low 25(OH)D_3_ serum levels (< median; Table 5). In logistic regression analyses, age, advanced liver fibrosis, presence of diabetes, and season of blood sampling were independent and significant predictors of low 25(OH)D_3_ serum levels (Table 5).

### Serum Concentrations of 25-hydroxyvitamin D and Treatment Outcome

Since 25(OH)D_3_ serum concentrations were only available in a subgroup of all treated patients included in this study (n = 269), we analyzed their relationship to treatment outcome separately from the above-described *CYP27B1* candidate gene study. In univariate analyses, no significant associations between SVR and 25(OH)D_3_ serum levels either as continuous variable (p = 0.13, OR = 0.98, 95% CI = 0.95–1.01), or 25(OH)D_3_ serum levels ≥10 ng/mL (p = 0.3, OR = 0.74, 95% CI = 0.43–1.30) and ≥20 ng/mL (p = 0.08, OR = 0.61, 95% CI = 0.36–1.10) were observed. Formally, these associations may be even interpreted as a statistical trend towards an inverse correlation between 25(OH)D_3_ serum levels and SVR after treatment with PEG-IFN-α and ribavirin. However, in multivariate models adjusted for other predictors of treatment outcome (age, sex, *IL28B* rs12979860 genotype, HCV genotype, HCV RNA levels, presence of diabetes, BMI, and liver fibrosis), 25(OH)D_3_ serum levels were clearly not associated with treatment outcome (p = 0.9 for 25[OH]D_3_≥20 ng/mL). These findings were similar when HCV genotype 1/4 or 2/3 patients were analyzed separately (data not shown).

## Discussion

The results of the present genetic validation study suggest, in line with our previously published findings [18], an association between the *CYP27B1-1260* promoter SNP rs10877012 and SVR to treatment of chronic hepatitis C with PEG-IFN-α and ribavirin. In the present study, this association was found only in patients with a poor-response *IL28B* genetic background, whereas *CYP27B1-1260* rs10877012 was not significantly associated with SVR in patients with good-response *IL28B* genotype. *CYP27B1-1260* rs10877012 is a functional polymorphism in the promotor of the 1α-hydroxylase, the enzyme required for the bioactivation of 25(OH)D_3_ to 1,25(OH)_2_D_3_ (calcitriol) [20]. It has been shown that the CC genotype of *CYP27B1-1260* rs10877012 impairs the expression of the 1α-hydroxylase, which results in reduced concentrations of bioactive vitamin D [18], [27]. Consistently, the CC genotype of *CYP27B1* is associated with poor response to interferon-α-based treatment of chronic hepatitis C in the present and in our previous study [18], as well as with the risk of bone disease or autoimmune disorders such as multiple sclerosis or type 1 diabetes [19], [20], [27], [29]. Importantly, 1α-hydroxylase is expressed not only in the kidney but also in inflamed tissue and even in immune cells, were it serves as a local, inducible producer of calcitriol [30]. Bioactive vitamin D is an important immune modulator, as for example T cells and macrophages crucially depend on calcitriol in various conditions [31]–[33]. Thus, one may speculate that the “poor-response” *CYP27B1-1260* rs10877012 genotype CC may result in lower local concentrations of calcitriol in the HCV-infected liver, resulting in reduced responsiveness to IFN-α or impaired adaptive immune responses. This may be especially relevant in patients with unfavourable *IL28B* genotype, who in general poorly respond to IFN-α.

The present study confirms that patients with chronic hepatitis C patients have a high prevalence of vitamin D insufficiency. However, in our study, 25(OH)D_3_ serum levels were not associated with treatment outcome in a subgroup of 269 patients with available baseline serum samples before antiviral treatment. In fact, 25(OH)D_3_ serum levels were even somewhat lower in patients who subsequently achieved SVR as compared to those who failed to respond to treatment. In two previous studies, including 167 and 211 patients treated with IFN-α-based therapy, a weak but significant correlation between 25(OH)D_3_ serum levels and SVR was observed [16], [34]. In our previous analysis we did not observe any significant association between 25(OH)D_3_ serum levels and SVR to IFN-α-based therapy in a cohort of 317 HCV genotype 1-infected patients, but a significant association in a cohort of 156 patients infected with genotype 2 or 3 [18]. Two studies in HCV-HIV-coinfected patients found no correlation between 25(OH)D_3_ serum levels and treatment outcome as well [35], [36]. The reasons for these discrepancies remain unclear at the moment, but apparently 25(OH)D_3_ serum levels cannot be considered as an established predictor of treatment outcome at the moment.

Importantly, it is well-known that 25(OH)D_3_ serum levels correlate poorly with calcitriol serum concentrations, and 25(OH)D_3_ serum levels are therefore not a suitable marker for bioactive vitamin D or vitamin D receptor signaling, especially not for local calcitriol levels during inflammatory conditions [24]. Thus, the lacking lack of an association between 25(OH)D_3_ serum levels and SVR may simply reflect the limited biological relevance of 25(OH)D_3_ serum levels. Unfortunately, there are no reliable methods to quantify serum levels of the bioactive vitamin D metabolite calcitriol, and the majority of clinical trials assessing the vitamin D status of patients focus on the calcitriol precursor 25(OH)D_3_ [24]. Therefore, despite the lack of an association between 25(OH)D_3_ serum levels and SVR, the replicated association between SVR and a functionally relevant genetic polymorphism in the vitamin D cascade, *CYP27B1-1260* rs10877012, suggests a role of vitamin D in the response to treatment of chronic hepatitis C. In line with this notion, we have recently identified an interaction between the vitamin D receptor and IFN-α-induced signaling through the Jak-STAT pathway, which results in a synergistic effect of calcitriol and INF-α on interferon-stimulated gene expression as well as on HCV replication *in vitro* (CML, MHH and DM, unpublished data). Therefore, the question as to whether optimization of the patients’ vitamin D status may be beneficial before or during antiviral therapy remains open.

In conclusion, the present study suggests a role of vitamin D metabolism in the response to treatment of chronic hepatitis C, especially in patients with poor-response *IL28B* genotype, but importantly 25(OH)D_3_ serum levels are not a reliable marker of treatment outcome.

### Limitations

Several limitations of our study have to be acknowledged. First, the association between *CYP27B1-1260* rs10877012 and SVR is statistically relatively weak, as it might be expected from the moderate impact of this variation on calcitriol synthesis [20], [27]. In line with this notion, we did not observe any significant association between *CYP27B1-1260* genotype and acute clearance from HCV infection in a cohort of 112 patients (data not shown). Therefore, *CYP27B1-1260* rs10877012 genotype does not appear to be a suitable marker for clinical decision making, and even larger sample sizes may be required to fully confirm the association between *CYP27B1* and SVR. Nevertheless, we believe that the importance of this genetic validation study lies in the identification of vitamin D signaling as an intrinsic player in IFN-α-based therapy of chronic hepatitis C. Second, 25(OH)D_3_ serum levels were available only for a subgroup of treated patients (n = 269), and significant associations might be identified in larger sample sizes. Furthermore, we cannot exclude a possible selection bias due the availability of serum in only a limited proportion of patients included in the primary analysis of *CYP27B1* genotype. Finally, incomplete datasets in some of our patients may be an additional source of bias, which represents a limitation inherent to cohort studies as compared to randomized controlled trials.
